# Exogenous Testosterone Enhances the Reactivity to Social Provocation in Males

**DOI:** 10.3389/fnbeh.2018.00037

**Published:** 2018-03-02

**Authors:** Lisa Wagels, Mikhail Votinov, Thilo Kellermann, Albrecht Eisert, Cordian Beyer, Ute Habel

**Affiliations:** ^1^Department of Psychiatry, Psychotherapy and Psychosomatics, Medical Faculty, Uniklinik RWTH Aachen University, Aachen, Germany; ^2^Institute of Neuroscience and Medicine 10, Research Center Juelich, Juelich, Germany; ^3^JARA-Institute Brain Structure Function Relationship, Research Center Juelich and RWTH Aachen University, Aachen, Germany; ^4^Institute of Pharmacology and Toxicology, Medical Faculty of RWTH Aachen University, Aachen, Germany; ^5^Hospital Pharmacy, University Hospital RWTH Aachen, Aachen, Germany; ^6^Institute of Neuroanatomy, Medical Faculty, RWTH Aachen University, Aachen, Germany

**Keywords:** aggression, testosterone administration, status hypothesis, challenge hypothesis, males, placebo effect

## Abstract

Testosterone affects human social behavior in various ways. While testosterone effects are generally associated with muscular strength and aggressiveness, human studies also point towards enhanced status–seeking motives after testosterone administration. The current study tested the causal influence of exogenous testosterone on male behavior during a competitive provocation paradigm. In this double blind, randomized, placebo (PL)-controlled study, 103 males were assigned to a PL or testosterone group receiving a colorless PL or testosterone gel. To induce provocation, males played a rigged reaction time game against an ostensible opponent. When participants lost, the opponent subtracted money from the participant who in return could subtract money from the ostensible opponent. Participants subjectively indicated anger and self-estimated treatment affiliation (testosterone or PL administration). A trial-by-trial analysis demonstrated that provocation and success during the repeated games had a stronger influence on participants’ choice to reduce money from the opponent if they had received testosterone. Participants who believed to be in the testosterone group were angrier after the experiment and increased monetary reductions during the task course. In line with theories about mechanisms of testosterone in humans, provocation is shown to be necessary for the agency of exogenous testosterone. Thus, testosterone reinforces the conditional adjustment of aggressive behavior but not aggressive behavior *per se*. In contrast undirected frustration is not increased by testosterone but probably interferes with cognitive appraisals about biological mechanisms of testosterone.

## Introduction

The influence of testosterone (T) on aggression has been studied across a variety of species. Animal research overall supports the assumption of increased aggression associated with high T plasma levels (Gleason et al., 2009). The basis of such a relationship has been unveiled via the *Challenge Hypothesis* (Wingfield et al., 1990) based on the observation that T plasma levels in male birds would rise as a function of social challenges. This in turn increased aggressive reactions. A second hypothesis, the *Status Hypothesis* was synthesized upon the *Challenge Hypothesis* later on, suggesting that T effects in humans depend on the challenge of the social status and that the direction of the effect will support status-seeking behaviors (Eisenegger et al., 2010). Thus, both prosocial and aggressive behaviors can be promoted via T depending on the context.

Different mechanisms, traditionally divided in organizational and activational effects, may explain the role of T in aggression. First, organizational effects of T in humans might determine the development of aggressive traits (Turanovic et al., 2017). Organizational effects are actions of steroid hormones which occur during early critical periods to organize neural pathways which could be responsible for certain behaviors such as aggression. While organizational effects occur early in life and are permanent, activational effects are transient and occur throughout life. Activational effects may thus influence human behavior via rapid changes in the neural circuit of aggression (Goetz et al., 2014). Despite of numerous studies investigating activational effects of T in the context of aggression and competition (Carré and Olmstead, 2015), causal evidence in humans is still rare. One reason for this is the predominantly correlational nature of earlier human studies supporting a weak but positive correlation of aggression with basal T (Archer et al., 2005). For instance, in inmates overt confrontations have been associated with higher salivary T (Dabbs et al., 1995). Critically, T might increase violent and aggressive behavior, however, it is equally possible that frequent aggressive acts increase T levels over time.

Thus, aggression research has further focused upon effects of exogenous T in humans (for an overview see Bos et al., 2012). While numerous studies investigated subtle effects of T on social-emotional behavior, initially primarily females were investigated due to the existence of an appropriate administration paradigm in females (Tuiten et al., 2000). Later on, studies were conducted in males mainly applying T via dermal administration (Zak et al., 2009; Cueva et al., 2015, 2017; Bird et al., 2016; Kopsida et al., 2016; Welling et al., 2016; Carré et al., 2017; Panagiotidis et al., 2017; Wagels et al., 2017a). Partly, studies investigated aggressive or antisocial behavior in males (Zak et al., 2009; Dreher et al., 2016; Kopsida et al., 2016; Carré et al., 2017; Cueva et al., 2017; Panagiotidis et al., 2017).

Two paradigms were most frequently applied to investigate the T-aggression relationship: The Ultimatum Game (UG) or the Point Subtraction Aggression Paradigm (PSAP). The UG represents a negotiation between two players. Usually, player one has a certain amount of money and is asked to make an offer to a second player sharing some of the money. The second player can accept the offer, which means that the deal is carried out, or reject the offer which means that both players will not receive any money. In the UG mostly rejections of low offers, which to some degree reflect social provocation, have been studied. While there is some evidence for T administration enhancing the likelihood of rejecting low offers (Zak et al., 2009) other studies neither support this in males nor females (Zethraeus et al., 2009; Eisenegger et al., 2010; Dreher et al., 2016; Kopsida et al., 2016; Cueva et al., 2017). Instead, in females sublingual T administration increased fair bargaining behavior (Eisenegger et al., 2010). Considering that fair behavior might avoid conflict—or social threat—the T effect here might be in line with the *Status Hypothesis*. Interestingly, females in the same study were less fair when believing to be in the T group. Cognitive appraisal about hormonal effects thus might be relevant and potentially contribute to the mixed findings that are reported in the UG. Nevertheless, underlining the assumption that T predominantly supports status-seeking behavior which depends on the context, males were shown to administer higher punishments to low offers but also higher rewards to generous offers in a modified UG (Dreher et al., 2016). Critically, antisocial behavior—rejecting an offer, or punishing the opponent—influenced the actual earnings of an individual. This conflict of reward-seeking and punishment or fairness motivation may be another reason for the divergent findings in the UG and impede the direct investigation of aggression.

Probably more closely investigating aggression, the effect of T administration has been studied during the PSAP several times. The PSAP involves an ostensible opponent who can steal points from the participant. The participant, on the other hand, can repeatedly choose between three buttons: A money button, a protection button or a counterattack button. Stolen points from attacking the opponent usually are not added to the participants’ earnings. First studies showed that long-term T administration of a supraphysiologic doses over several weeks (2 and 6) increased aggressive responses (the number of attack button presses) towards social provocation in the PSAP (Kouri et al., 1995; Pope et al., 2000). Newer findings which tested the effect of a single T administration in males did not confirm enhanced aggressive reactions towards social provocation after T administration (Carré et al., 2017). However, the authors found that T increased aggressiveness in men who were highly dominant or low in self-control. Again, the effect of T on the reaction towards a social challenge seems to depend on individual (status-seeking) motives. A disadvantage of the PSAP is that provocation frequency of the ostensible opponent depends on the participant’s behavior (frequent protect or attack decisions will result in less provocations as the program usually blocks provocations for a certain time after these decisions). Although researchers try to control for task variability, the initial situation participants are confronted with might differ strongly and thereby disguise effects of T.

In order to gain a better understanding of how exogenous T influences males behavior during a provocation task, we applied a modified version of the Taylor Aggression Paradigm (TAP; Giancola and Parrott, 2008). We primarily aimed to circumvent two limitations of the above reviewed paradigms: First, earnings should be independent from the punishment decision of the participant; second, predefining the opponent’s behavior, provocation situations were fixed. During the TAP participants play repeated rounds of reaction-time games against an ostensible opponent. Both players can ostensibly punish the other player by reducing money, when winning the round which is not added to their actual earnings. Previous research showed that participants in this task act with “*tit-for-tat*” like behavior towards the punishment of the feasible opponent (Krämer et al., 2007). In detail, this means that participants adjust their punishment levels to the preceding provocation. Such behavioral adjustments might reduce conflict potential and protect the social status.

Assumptions of the current study were based on the *Challenge Hypothesis* and the *Status Hypothesis*. First, exogenous T should enhance punishment behavior during the modified TAP due to the social competition. Especially losing would constitute a social challenge thus promoting aggressive behavior. Moreover, we expected that exogenous T would increase *tit-for-tat* like behavior compared to the placebo (PL) group in order to gain a high social status. Since this has not been investigated before, we also investigated in an exploratory way the temporal course of punishment behavior comparing T and PL.

A secondary goal of the study was to investigate if aggression is related to the subjective belief of having received T (or PL). Since a previous study demonstrated that the belief to have received T leads more rejections in the UG (Eisenegger et al., 2010), we expected that individuals who believe to be in the T group would react more aggressively independent of the provocation.

## Materials and Methods

### Sample

The study included 103 male participants recruited in Aachen via online advertisements and postings. For their participation, participants received a fixed amount of 70 Euros and additionally the money they won in two further paradigms they performed in the study. All participants had normal or corrected vision, no contraindications for magnetic resonance imaging (MRI), and no history of traumatic brain injury, psychiatric or neurological illness and were right handed (according to Oldfield, 1971). Participants were between 18 years and 35 years old (*M* = 24.17, SD = 3.76). Ethnicity was not explicitly ascertained. A more detailed description of the sample is reported elsewhere (Wagels et al., 2017b). Written and informed consent was obtained from all participants in accordance with the recommendations of the Declaration of Helsinki. After the scanning session, participants were fully debriefed about the study aims and the cover story around the paradigm. The study was approved by the internal ethics committee of the RWTH Aachen medical faculty and was not evaluated as clinical trial. We therefore did not register as a randomized controlled trial in any official online register.

### Procedure

**Figure 1:**
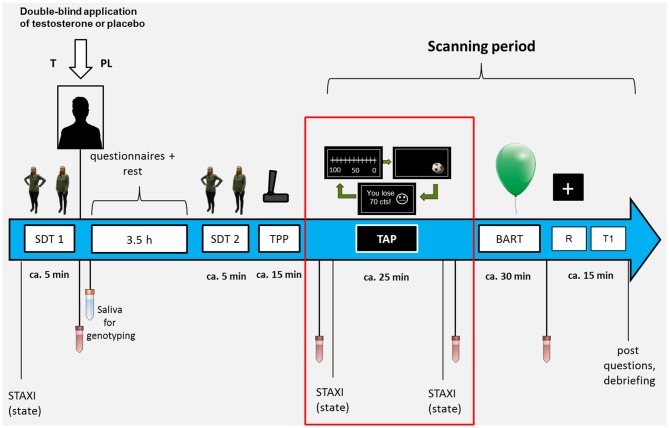
The figure presents an overview on the course of the complete study procedure. SDT, Stop distance paradigm; TPP, technical provocation paradigm; TAP, Taylor Aggression Paradigm; BART, Balloon Analogue Risk Task; R, resting state; T1, anatomical scan.

The TAP was part of a large study including several tasks related to aggression (non-social and social aggression) and risk-taking of which two were performed in an MRI environment (for an overview see Figure 1). The TAP was the first task of the scanning session. One major aim of the complete study was to investigate the interaction of T administration and genetic variability in the serotonergic system (MAOA VNTR polymorphism, and serotonin transporter polymorphism) regarding neural responses. Results concerning this gene-hormone interaction have been published for the risk task (Wagels et al., 2017b). In order to have a clear focus on the influence of T administration in a new social aggression paradigm this manuscript only presents behavioral data and will focus on the gene-hormone interaction on a neural level elsewhere.

In order to reach a stable hormonal level at the baseline measurement, sessions started between 12:00–14:00 and took about 6.5 h. After taking a first blood sample to determine baseline serum levels, participants received either 5 g Testim™ corresponding to 50 mg T, or an equivalent amount of sonography gel (PL). The gel was applied on the upper part of the back and the shoulders of participants by a blinded experimenter. Participants performed several short tasks, filled out personality questionnaires, provided saliva samples for genotyping analysis and had about 1 h break before the scanning session.

To improve the credibility of the aggression paradigm, before the scanning session individuals were introduced to an ostensible male opponent who was supposedly guided to a separate test room. Before and after the task blood samples were taken to test for task effects on T plasma levels. The task was followed by another experimental task on risk-taking, a resting-state measurement and an anatomical scan. After scanning participants were asked if they believed to have received T or PL.

### Task: Modified Taylor Aggression Paradigm (TAP)

**Figure 2:**
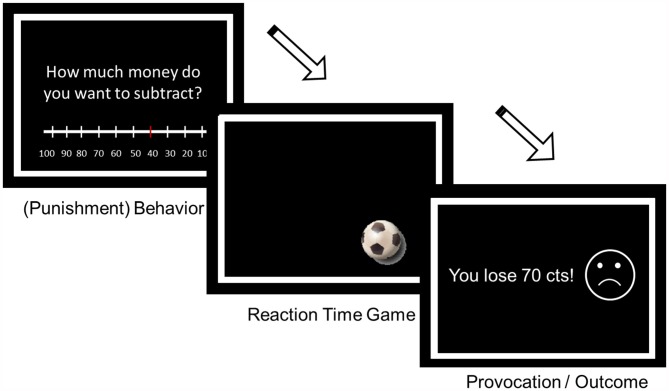
The task is split in three main parts: during the decision period, the participant can decide how much money he wants to subtract from the opponent; during the game period the participant plays a reaction time game against an ostensible opponent; during the feedback period, the participant sees if he won or lost and how much money he lost due to the ostensible opponents decision. The last period was assumed to influence the behavior in the following trial.

The TAP is a well-validated aggression task (Giancola and Parrott, 2008) usually disguised as a reaction-time game against a real opponent (for the modified version applied here, see Figure 2). Participants were instructed to react as fast as possible to a target (fast moving soccer ball) appearing in any corner of the screen. If they were faster than their opponent they would win 50 cents, otherwise they would lose 0–100 cents. The amount of money they would lose was ostensibly determined by their opponent on an 11-ary scale and was presented at the end of the trial in an actually predefined pseudo-randomized order. Individuals could decide how much money they would reduce from their opponent in case they would win at the beginning of each trial. It was stated clearly, that neither the opponent nor the participant would earn the money they subtracted but only the 50 cents they earned in win trials. Monetary reductions thus were not related to reward but consistent with the definition of aggression as a goal-directed behavior with the intent to harm another individual who is motivated to avoid such a treatment (Baron and Richardson, 1994).

In total, there were 54 predefined lost trials (23 high provocation trials: 80–100 cents, 25 low provocation trials: 0–20 cents, 6 medium provocation trials: 30–70 cents) and 30 trials in which they won (always 50 cents). Minor variations could emerge when individuals’ reaction time was below 600 ms. In these cases, individuals lost the trial followed by a medium provocation trial (50 cents). Overall, the paradigm lasted 25 min.

An important advantage of the current task compared to the PSAP is the control of provocations which can be gradually modified and which are the same for each participant. Moreover, since the course of the task is the same for each participant it is possible to study both adaptive behavioral changes (punishment adjustments depending on the strength of the provocation) and accumulative frustration (a time-dependent increase of punishment levels) and if these are influenced by the administration of T.

### Hormonal Levels

T and cortisol (C) levels were analyzed with immunologic *in vitro* quantitative determination of T/C in human serum and plasma (Electrochemiluminescence immunoassay, ECLIA; Roche^®^ Diagnostics GmbH, Mannheim, Germany)^1^. In order to verify the treatment success and task influence on T levels, a repeated-measures ANOVA was performed with time as within-subject variable and treatment group as between-subject variable. The same procedure was performed for C levels.

### Behavior and Emotions

To investigate task-related behavior, we fitted a general linear model on a trial-by-trial basis, which aimed at predicting aggressive behavior of the volunteer using the amount of money he subtracted from his ostensible opponent at each single trial as surrogate. Hence, the amount of money participants reduced in a specific trial (trial x, where x denotes the trial number) was the dependent variable reflecting the participants’ aggressive behavior within the trial. As predictors, we included the outcome of the game (win = 1 vs. lose = 0) in the preceding trial (x − 1) and the amount of money reduced by the opponent (0–100) in the preceding trial (x − 1) as well as the trial number x (1–84) modeling linear temporal shifts in aggressive behavior. An intercept was also included in order to account for individual aggression levels across the whole task. All parameters were estimated for each participant and included in a full factorial analysis with the between-subject factor treatment (T, PL). We also included the subjective treatment believe (bT, bPL) as covariate to control for a potential influence. Outliers were excluded if the deviation was more than 2 standard deviations above the mean (Supplementary Figure S1).

Parallel to the behavioral responses, emotional effects of the task were tested measuring state anger. Therefore, the difference score (post task—pre task) of the State-Trait-Anxiety Inventory (STAXI, Schwenkmezger et al., 1992) was estimated. Treatment group was added as between-subject factor and the subjective belief about the received treatment was included as covariate.

### Additional Exploratory Analyses

In order to test the relationship of task related anger and task related aggression, individual parameter estimates of the model (temporal course, outcome, provocation, intercept) were included in a step-wise regression analysis. As dependent variable, the STAXI state score (post task-pre task) was added.

Assuming that the relationship of anger and aggression could be influenced by treatment and treatment belief, significant parameters were applied to a moderated moderation model. In detail, we tested a model in which aggression parameters were applied as dependent variable, the anger increase as predictor variable and treatment as well as treatment belief as moderator variables. This procedure was performed with the PROCESS tool of SPSS (Hayes, 2012) applying model 3. This model assumes a three-way interaction of treatment, treatment belief and anger.

## Results

### Group Characteristics

Participants belief to have received PL or T was independent of the received treatment, $X_{\left(1,N\;=\;92\right)}^{2}$ = 0.25, *p* = 0.397, $\eta_{\text{p}}^{2}$ = 0.052, see Table 1. The other comparisons of trait aggression between treatment groups did not indicate any group differences, *F*_(4,95)_ = 0.32, *p* = 0.880, $\eta_{\text{p}}^{2}$ = 0.012.

**Table 1:** Distribution of subjective treatment believe and actual treatment group.

	T	PL
bT	*n* = 12	*n* = 12
bPL	*n* = 38	*n* = 30

### Hormone Plasma Levels

**Figure 3:**
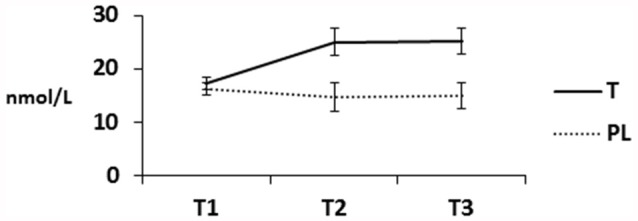
Serum T in nmol/L separately for participants of the testosterone (T) and placebo (PL) group (T1 = baseline before gel administration, T2 = ~4 h later before the aggression task, T3 = ~4.5–4.75 h later). Means and standard errors are presented.

The analysis included 97 participants since blood samples at T3 could not be gathered of six participants. T plasma levels (Figure 3) differed between groups, *F*_(1,95)_ = 14.38, *p* < 0.001, $\eta_{\text{p}}^{2}$ = 0.131, between measurement time points, *F*_(1.30,94)_ = 5.90, *p* = 0.01, $\eta_{\text{p}}^{2}$ = 0.058, and as a function of group by measurement time point, *F*_(1.30,94)_ = 45.71, *p* < 0.001, $\eta_{\text{p}}^{2}$ = 0.325. T plasma levels differed between the T and PL group at time 2 (*p* < 0.001) and time 3 (*p* < 0.001), but not at baseline (*p* = 0.63). In the PL group, T plasma levels significantly decreased from T1 to T2 (*p* = 0.002), and T1 to T3 (*p* = 0.009), but did not differ between T2 and T3 (*p* = 0.100). In the T group, T plasma levels increased from T1 to T2 (*p* < 0.001), from T2 to T3 (*p* = 0.002), and from T1 to T3 (*p* < 0.001). C levels decreased over time in both groups, *F*_(1.5,93)_ = 54.02, *p* < 0.001, $\eta_{\text{p}}^{2}$ = 0.365, but did not differ between groups (*p* = 0.170).

### Behavior and Emotions

**Figure 4:**
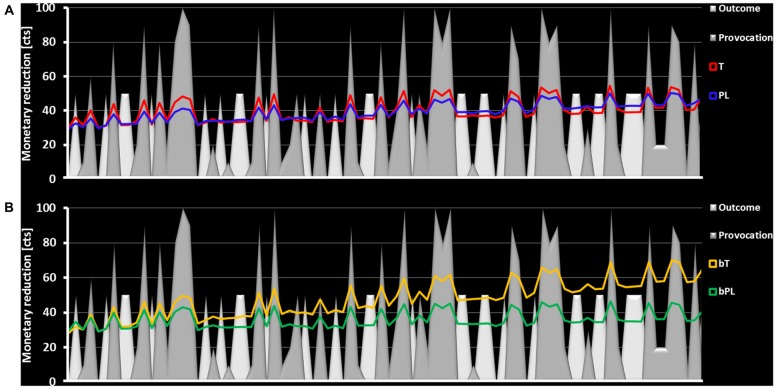
The figure presents the time course of the task. The provocation factor is represented via gray planes with the height representing the amount of the reduced money. The outcome factor is represented by white planes with the height representing the reward value (continuously 50 cents). **(A)** The money subtraction in the T group (red) follows the provocation and outcome more strongly than in the PL group (blue) as here observed via higher amplitudes following gray and white planes. **(B)** Participants who believed to have received T (yellow) demonstrate an increased money subtraction over time compared to those who believed to have received a PL (green).

The analysis included 88 participants in total due to outliers and missing information about believing to have received a PL or testosterone gel. Task related aggressive behavior, measured by the amount of subtracted money from the ostensible opponent, could be significantly explained by prior provocation, *F*_(1,85)_ = 36.78, *p* < 0.001, $\eta_{\text{p}}^{2}$ = 0.302, prior outcome, *F*_(1,85)_ = 19.83, *p* < 0.001, $\eta_{\text{p}}^{2}$ = 0.185, but not by the time course of the task when controlling for subjective believe in having received treatment, *F*_(1,85)_ = 2.66, *p* = 0.087, $\eta_{\text{p}}^{2}$ = 0.030. Significant differences of the T and PL group were noticed for provocation, *F*_(1,85)_ = 5.61, *p* = 0.020, $\eta_{\text{p}}^{2}$ = 0.062, and outcome parameters, *F*_(1,85)_ = 11.90, *p* = 0.001, $\eta_{\text{p}}^{2}$ = 0.120 (Figure 4A). The mean effect of provocation in the T group was larger than in the PL group (T: *M* = 0.18, CI [0.13; 0.23] PL: *M* = 0.09, CI [0.04; 0.15]) and the mean effect of outcome was smaller in the T group than in the PL group (T: *M* = −7.76, CI [−10.17; −5.36] PL: *M* = −1.49, CI [−4.19; 1.20]). Please note that a negative effect for outcome represents that losses coded with 0 lead to higher monetary reductions than winnings coded with 1. The intercept which reflected the individual estimate of overall task aggression did not differ significantly between the T and PL group, *F*_(1,85)_ = 0.05, *p* = 0.830, $\eta_{\text{p}}^{2}$ = 0.001. Finally, the time course was significantly influenced by the believe to have received T or PL, *F*_(1,85)_ = 8.72, *p* = 0.004, $\eta_{\text{p}}^{2}$ = 0.093. In order to follow-up this covariate effect, a *t*-test for independent samples was performed. The *post hoc* test indicated that bT participants, compared to bP participants, became more aggressive during the task, *t*_(85)_ = 2.95, *p* = 0.004 (Figure 4B).

**Figure 5:**
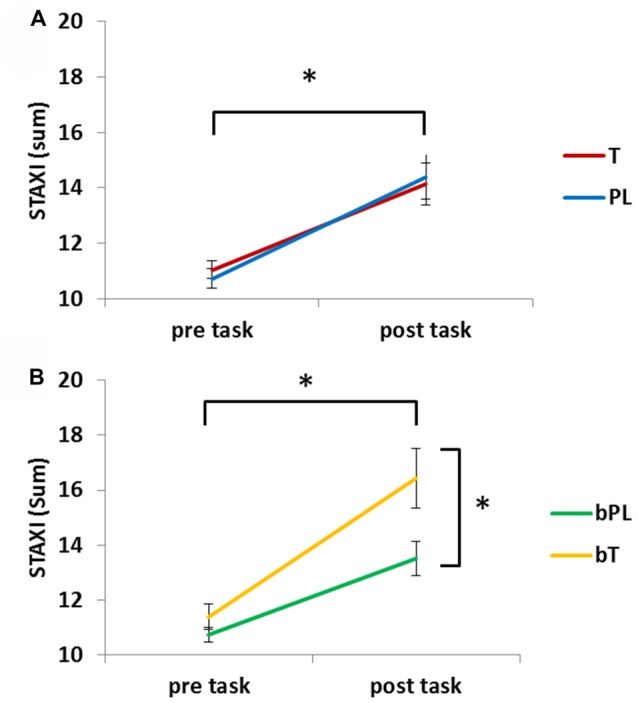
Means and standard errors for anger ratings [State-Trait-Anxiety Inventory (STAXI)] before and after the task are presented. **(A)** Anger increased significantly in the testosterone (T) and placebo (PL) group. **(B)** Anger increased significantly more in participants that believed to have received testosterone (bT) than those who believed to have received placebo (bPL). *Significant effect (*p* < 0.05).

The analysis of anger ratings included 91 participants due to missing information about believing to have received a PL or testosterone gel. In order to evaluate if the task induced negative emotions, state anger (STAXI) pre and post task was compared (Figure 5). The repeated measures ANCOVA revealed a significant effect of task with increased anger after the task, *F*_(1,88)_ = 30.00, *p* < 0.001, $\eta_{\text{p}}^{2}$ = 0.254. There was no significant difference between the PL and T group (*p* = 0.961) and no interaction of task and treatment (*p* = 0.513), however, the interaction of task and the covariate treatment belief was significant, *F*_(1,88)_ = 4.00, *p* < 0.030, $\eta_{\text{p}}^{2}$ = 0.052 and there was a main effect of the covariate treatment belief, *F*_(1,88)_ = 4.64, *p* < 0.034, $\eta_{\text{p}}^{2}$ = 0.050. *Post hoc* analyses demonstrated that the bT group was overall more angry, *F*_(1,89)_ = 4.69, *p* = 0.033, $\eta_{\text{p}}^{2}$ = 0.05 (bT: *M* = 13.91 ± 0.71, bP: *M* = 12.13 ± 0.46) while groups did not significantly differ before the task (*p* = 0.233). Thus, after the task, the bT group was significantly more angry than the bP group, *F*_(1,89)_ = 5.40, *p* = 0.022, $\eta_{\text{p}}^{2}$ = 0.057 (bT: *M* = 16.44 ± 1.1, bP: *M* = 13.51 ± 0.63).

### Relationship Anger and Aggression

First, associations of emotions and behavior within the social context were tested. Including all task-related model parameters in a step-wise regression model, the temporal course of aggressive behavior and the aggression level were selected as significant predictors for task related anger increase, *F*_(2,96)_ = 11.47, *p* < 0.001. Both predictors were positively associated with the anger increase meaning that a temporal aggression increase during the task predicted higher post task anger, *b* = 4.66 ± 1.03, *p* < 0.001, and similarly a higher general aggression level (intercept) predicted higher post task anger, *b* = 0.030 ± 0.012, *p* = 0.020. Neither outcome related behavior nor provocation related behavior were significant predictors of task related anger and were thus excluded for the model estimation.

Two moderated moderation analyses were performed separately for the temporal aggression parameter and the intercept. The intercept-model was not significant, *R* = 0.16, *p* = 0.957. The temporal-model was significant, *R* = 0.56, *p* < 0.001. Significant model contributions are given in Table 2. Notably, the interaction of treatment, treatment belief and anger was not statistically significant—but there was a trend for the interaction of treatment belief and anger (*p* = 0.060) as well as treatment and treatment belief (*p* = 0.051). Conditional effects indicated that the relationship of anger and temporal aggression increase was significant for the PL group both for bT, *b* = 0.028 ± 0.01, *p* = 0.036 and bPL, *b* = 0.074 ± 0.03, *p* = 0.004. In the T group, the relationship was only significant for bT, *b* = 0.069 ± 0.02, *p* = 0.002 but not for bPL, *b* = 0.022 ± 0.01, *p* = 0.114.

**Table 2:** Moderated moderation on the temporal aggression course.

	Coeff	se	*t*	*p*	LLCI	ULCI
Constant	0.11	0.03	3.49	0.001	0.048	0.175
Treatment	0.10	0.07	1.50	0.137	−0.032	0.225
Anger increase	0.04	0.01	3.96	<0.001**	0.018	0.054
Treatment × anger increase	0.01	0.02	0.56	0.575	−0.026	0.047
Treatment belief	0.19	0.08	2.48	0.015**	0.037	0.341
Treatment belief × anger increase	0.05	0.02	1.91	0.060*	−0.002	0.095
Treatment × treatment belief	−0.30	0.15	−1.98	0.051*	−0.61	0.001
Treatment × treatment belief × anger increase	0.01	0.05	0.28	0.779	−0.087	0.115

*trend level p < 0.10; **significant p < 0.05.

## Discussion

The current study investigated the causal role of T on males’ punishment behavior following social provocation. Exogenous T led to an increased *tit-for-tat* behavior (Krämer et al., 2007) of male participants: Compared to the PL group, the T group responded with larger punishments after high but smaller punishments after low provocations and wins. Replicating similar findings, we demonstrate that T is context sensitive, likely supporting status-seeking behaviors (Dreher et al., 2016). The T effect does not seem to be a result of overall enhanced frustration since post-task anger and overall aggression levels were unaffected by T administration. However, participants who believed to have been treated with T were angrier after the task and dispensed increased punishment levels to their apparent opponent.

### Provocation as Necessary Trigger for Aggressive Reactions

Provocation as described via money reduction by an ostensible opponent or by losing a costly competition against this opponent, in the current study led to higher punishment of the opponent. Participants did not profit from monetary reductions but knew that these reductions would diminish the earnings of their opponent. Thus reducing money from the ostensible opponent had the intention to harm another individual who is motivated to avoid this harm—defining an aggressive act (Baron and Richardson, 1994). The punishments participants chose may quantify the harm participants want to inflict on their opponent and thus characterize their aggressiveness. We assessed aggressiveness in relation to several factors, the outcome of the reaction-time games, the provocation by the ostensible opponent, and the time course of the task. Aggressive behavior did not differ between T and PL measured as general level, and the time course of aggressive actions was comparable in both groups. Thus, we conclude that T administration did not affect aggressiveness *per se*.

Aggressiveness of participants that had received T more strongly varied towards the provocation of the opponent and the game outcome compared to participants in the PL group. T thus did not affect overall aggression but made participants more sensitive for contextual changes. Relative to the PL group participants acted with a stronger *tit-for-tat* behavior, acting less aggressively in low but more aggressively in high provoking situations. Aggressive reactions especially if oriented towards a provoking opponent may constitute retaliation. T administration thus seems to shift the need for retaliation: In low provoking situation this need is rather reduced but in high provoking situations it increases.

Considering that overall aggression did not differ but instead behavior more strongly followed the opponents’ provocation, this may provide some support for the *Status Hypothesis*. Assuming that losses as well as high provocations would constitute a status attack, T administration increases retaliatory behavior especially in situations in which the social status of males is endangered. Interestingly, it concerns about the social status seem to drive retaliatory behavior more so in men than in women (Geniole et al., 2015). We cautiously suggest that T might be an underlying factor for such gender-specific motivations of retaliating provocative acts. Status-seeking motives as underlying factor for a context dependent enhancement or reduction of aggressiveness, would be in line with numerous studies explaining bidirectional T effects in social interactions (Eisenegger et al., 2011).

Research of the past years demonstrated that T effects underlie both individual and contextual characteristics. With regard to aggression, especially high individual trait dominance may be needed to observe enhanced aggression as a non-genomic effect of increased T (Carré et al., 2017). It may be speculated that highly dominant males would have reacted with higher punishments throughout the TAP, but this has to be confirmed in future studies. We here find some evidence for the influence of the context, which has mostly been observed in economic gambling paradigms such as the UG (Eisenegger et al., 2010; Dreher et al., 2016). In contrast to the UG, aggressive decisions in this study were neither costly nor rewarding for the individual and thus might not be driven by the motivation to earn (or lose) more money via high punishments.

While the findings replicate the context effect, it remains open if T modified the perception of the context or the decision of how to act in a corresponding context. Several studies suggest that exogenous T modifies the perception of social threat (van Honk et al., 2000; Wirth and Schultheiss, 2007; Wagels et al., 2017a). Neural processing of reward and social threat seems to be altered under T (Hermans et al., 2008, 2010; Radke et al., 2015). In turn this might produce a shifted sensitivity to reward and punishment as suggested before (van Honk et al., 2004). T administration in this study thus might have increased the feeling of being treated fairly in low provoking situations and the feeling of being treated unfairly in high provoking situations. Partly this may be supported when including the data of the previous experiment in the non-social environment (see Supplementary Table S1 for further details). Anger reactions in the non-social provocation task towards provocation, corresponded to the *tit-for-tat* behavior in the social aggression task. While, due to time constraints, specific emotional reactions to provocation and outcome could not be assessed, the data of the non-social context provide some support for an enhanced emotional reaction specifically to provocation which might reflect a shift in the perception under T administration.

On the other hand, it is still possible that the perception of fairness remained stable comparing T and PL groups, but the decision how to react in such a more or less fair situation shifted. While to our knowledge no study explicitly investigated this question regarding activational effects of T, organizational effects seem to alter punishing decisions without affecting the perception of the situation (Ronay and Galinsky, 2011).

Though the current results are in line with the *Challenge Hypothesis* (Wingfield et al., 1990), they emphasize the need of a precise characterization of the challenge. The decision of what is perceived as a challenge might be highly individual and context sensitive and is probably driven by status motives.

### Success vs. Failure

The influence of the competition outcome in the current paradigm has to be interpreted with caution, since all lost trials were confounded with provocation. Primarily, the observed effect of enhanced aggression after lost compared to won trials might be related to the provocation aspect since there was no provocation in the win trials. Nevertheless, the effect may also refer to the game outcome itself (e.g., participants after T administration simply act less aggressively after winning a competition). Competitive situations can modulate T levels (Geniole et al., 2017) and T levels can influence the willingness to engage in future competitions (Carré et al., 2013). In women, high T levels after winning a competition can predict prosocial behavior (Casto and Edwards, 2016). Future tasks should separate outcome and provocation phases to assess complete independent effects.

### Expectation Effect

While T administration may promote a *tit-for-tat* behavior but not aggressiveness *per se*, such anger driven reactions may be triggered by subjective beliefs about T. Similar to the current results, the subjective belief about the effects of T has been shown to promote antisocial egoistic bargaining behavior previously (Eisenegger et al., 2010). While not all studies observed assumed stereotypical behavior due to treatment expectations (Dreher et al., 2016; Cueva et al., 2017) some demonstrated that the belief to have received a steroid can increase physical performance and aggressive behavior (Björkqvist et al., 1994; Maganaris et al., 2000).

In the current study, we observed separate effects of T and the belief about the received treatment. We suggest that the belief in having received a T treatment is associated with stronger frustration as reflected by higher anger ratings after the task and also increasing aggressiveness. This may be a result of a conscious cognitive attribution process. On the one hand, believing that T increases aggression may work as a self-fulfilling prophecy and thereby make participants act more aggressively. On the other hand, attributing antisocial behavior to biological mechanisms may be a self-serving coping strategy excusing socially undesirable behavior. Notably, such a following explanation is rather unlikely since participant that believed to have received T indicated that they were angrier after the task and thus were not able to improve their coping. Possibly, the belief about having received a PL may attenuate the frustration elicited by the task. Interestingly, more participants believed to have received PL gel (66%). Possibly, participants expected physiological or behavioral changes if they received the testosterone gel. When they did not realize any changes they might have concluded to have received the PL gel. The results on the association of treatment belief and anger or aggressiveness therefore are not unambiguous: Either, an expectation might have elicited anger and aggression, or the retrospective evaluation of the behavior might have led to a conclusion about the treatment. Nevertheless, the results underline the importance of the cognitive influence which can be created via associations with the treatment. Thus, it is highly necessary to assess beliefs about treatment. Moreover, studies that systematically investigate a PL effect of T are needed to avoid interactions of the actual treatment and expectations.

### Limitations

The current study investigated a homogenous sample of young, healthy, male participants. It remains unclear if the results can be generalized to other populations e.g., females. Currently, administration studies are confronted with the difficulty of different permissions for males and females and the comparability of the T increases due to biological endowment.

The investigation of aggression is challenging due to many problems: Aggression is mainly defined as a social act and hence requires a social partner. For organizational purposes, the actors mimicking the opponent were different (all young male) persons possibly influencing the perception of the social communication. However, the random actor variance should not result in a systematic bias. In order to improve the credibility, the study was planned as between-subjects design not as a within-subject cross-over design which would be the preferred method to enhance power and reduce interindividual influences. The task was performed in a MRI environment limiting spatial and temporal freedom potentially inhibiting aggression.

## Conclusion

T administration does not generally increase aggression *per se*, but increases *tit-for-tat* behavior: The higher the provocation the higher the punishment, the lower the provocation, the lower the punishment. Supporting the *Status Hypothesis*, this again underlines the context sensitive effectiveness of exogenous T. In contrast, the belief to have received T might actually enhance frustration and leads to an increase of aggressive behavior. Alternatively, the assumption of having received T might provide a retrospective explanation and excuse of usually socially undesirable behavior.

## Author Contributions

LW was involved in designing the study, analyzing the data, performing the literature research and drafting the first version of the manuscript. MV was involved in designing the study, analyzing the data and correcting the manuscript. TK was involved in analyzing the data, and correcting the manuscript. AE and CB were involved in designing the study and correcting the manuscript. UH was involved in designing the study, interpretation of the data and correcting the manuscript.

## Conflict of Interest Statement

The authors declare that the research was conducted in the absence of any commercial or financial relationships that could be construed as a potential conflict of interest.

## Footnotes

^1^www.roche-diagnostics.com

## Supplementary Material

FIGURE S1 Mean values of aggressiveness separated for preceding provocation levels for the four outliers. Abnormal responding is mainly shown after medium provocation (indicated within the red frame).
