# Supplementary material for: Exogenous Testosterone Enhances the Reactivity to Social Provocation in Males
Source: Front Behav Neurosci. 2018 Mar 2;12:37. doi: 10.3389/fnbeh.2018.00037 (PMC5840258; doi:10.3389/fnbeh.2018.00037)
Supplement: Supplementary file 1 [file Data_Sheet_1.docx]

**Supplementary Material**

**Background**

As a post-hoc analyses, we aimed to compared the results of the non-social aggression paradigm and the social aggression paradigm. For the comparison emotional assessments and behavioral measures were compared. The non-social aggression paradigm or Technical Provocation Paradigm (TPP) was part of the current study and performed prior to the modified TAP. It aimed at testing the effects of provocation without including any other participant.

In order to provide an overview about the non-social aggression paradigm (TPP), we will provide a short description of the task and the dependent variables (for a more detailed description please refer to (Panagiotidis *et al*, 2017). Moreover, we would like to state why previous samples included only a subsample of the here reported sample. We include the complete sample of 103 individuals while in the previous manuscript about the non-social aggression paradigm only a subsample (90 participants) was investigated. The reason for publishing the results of a subsample was that the behavioral paradigms (see Panagiotidis *et al*, 2017; Wagels *et al*, 2017a) were part of doctoral theses which were planned with smaller samples. However, both paradigms were continuously included during the subsequent measures to maintain a comparable experimental setting for all participants. This allows a comparison of the modified TAP and the TPP including all participants here.

**Technical provocation paradigm (TPP)**

In the TPP, individuals were instructed to direct a moving ball into a barrel by pulling a joystick in order to win gold coins (virtual money). Applying a block design, there were two conditions, a control block and a provocation block. In the control block the outcome (hit vs. miss) was determined solely by the participants’ performance. In the provocation block the program was manipulated so that it would not react to pulling the joystick in several trials and in consequence participants missed the reward. In addition these provocation trials were followed by a message: “Please move the joystick!”.

Within the TPP emotions (specifically anger) were measured applying emotional self-ratings on a five-point Likert-like scale (Schneider *et al*, 1994). Furthermore, maximal joystick deflections of each trial were averaged per block indicating an implicit measurement of impulsive aggressive behavior.

**Statistical comparisons**

Emotional measures of the social and non-social aggression paradigm were exploratory compared via Pearson correlations (two tailed at α= .05). We included the difference score of anger (ESR) in the TPP (provocation – control), and the difference score of the anger rating (STAXI) in the modified TAP (post task-pre task). Moreover the difference score of the joystick deflection (provocation – control) and the task parameters of the modified TAP (provocation, outcome, temporal change and intercept) were included.

**Results**

Pearson correlations are presented in Table 1. In short, the emotional reaction in the TPP is related to the behavioral adjustments to provocation and outcome in the TAP. Increased anger reactions in the TPP were associated with higher aggression levels in the TAP (r = .29, p = .003), larger differences in the reaction towards provocation (r =.29, p = .003) and winning or losing the game (r = -.22, p = .028). The anger increase during the TAP was also related to the anger rating during the TPP - both in control (r =.35, p <.001) and provocation blocks (r =.33, p =.002). Anger in the TPP control condition was related to anger before (r = .31, p < .002) and after the TAP (r = .42, p < .001). The increasing aggression during the TAP (temporal course) was not related to any measures of the TPP and the joystick deflection in the TPP was not related to any measures in the TAP.

Table 1: Pearson correlations of emotions and behavior in the non-social (TPP) and social (TAP) aggression paradigm

|  | TPP anger _(diff)_ | TPP an-ger _(contr.)_ | TPP anger _(prov.)_ | TPP Joystick _(diff)_ | TAP anger _(post-pre)_ | TAP anger _(baseline)_ | | TAP anger _(pre)_ | TAP anger _(post)_ | | | TAP _temporal course_ | TAP  _outcome_ | | TAP _prov._ |
| --- | --- | --- | --- | --- | --- | --- | --- | --- | --- | --- | --- | --- | --- | --- | --- |
| TPP anger _(diff)_ |  |  |  |  |  |  |  | | |  |  | |  |  | |
| TPP anger _(control)_ | **-.44**** |  |  |  |  |  |  | | |  |  | |  |  | |
| TPP anger _(provocation)_ | **.73^**^** | **.29^**^** |  |  |  |  |  | | |  |  | |  |  | |
| TPP Joystick _(diff)_ | -.18 | **.23^*^** | -.03 |  |  |  |  | | |  |  | |  |  | |
| TAP anger _(post-pre)_ | .04 | **.35^**^** | **.31^**^** | -.05 |  |  |  | | |  |  | |  |  | |
| TAP anger _(baseline)_ | -.02 | .18 | .12 | .10 | .14 |  |  | | |  |  | |  |  | |
| TAP anger _(pre)_ | -.03 | **.31^**^** | **.20^*^** | .15 | .19 | **.53**** |  | | |  |  | |  |  | |
| TAP anger _(post)_ | .02 | **.42^**^** | **.34^**^** | .01 | **.93^**^** | **.32^**^** | **.55^**^** | | |  |  | |  |  | |
| TAP temporal course | -.16 | .11 | -.09 | .11 | **.24^*^** | .18 | .12 | | | **.27^**^** | |  |  |  | |
| TAP outcome | **-.21*** | .01 | **-.22** | .12 | -.05 | **-.24*** | **-.26*** | | | -.14 | | -.02 |  |  | |
| TAP provo-cation | **.28^**^** | -.02 | **.29^**^** | -.07 | .03 | .12 | .08 | | | .06 | | .11 | **-.63**** | |  |
| TAP intercept | .13 | .13 | **.24^*^** | -.01 | .17 | -.03 | .02 | | | .15 | | **-.25*** | -.01 | | .01 |

* p <.05; ** p<.001

**Discussion**

The comparison of the social and non-social aggression paradigms show that aggressive behavior within the social provocation paradigm is related to frustration effects both in the social and non-social provocation paradigm. It is especially interesting that individuals who reacted with increased anger in the non-social context also seem to show stronger reactions towards the provocation and the game outcome in the social context. These measures were shown to be affected by testosterone administration (for the TPP see Panagiotidis et al. 2017).

Taken together, the findings in the social and non-social aggression task underline the assumption that either social or non-social provocation is a necessary component for an effect of testosterone. Importantly, the effect seems to be specific and does not relate to measures which rather assess general tendencies (such as the joystick reflection, which rather correlated with anger in control conditions) or general aggression levels. Instead T induces a relative change in aggressive behavior. Possibly T induced shifts in general aggression tendencies are more strongly influenced by traits such as impulsivity or dominance (Carré *et al*, 2017).

**References**

Carré JM, Geniole SN, Ortiz TL, Bird BM, Videto A, Bonin PL (2017). Exogenous Testosterone Rapidly Increases Aggressive Behavior in Dominant and Impulsive Men. *Biol Psychiatry* **82**: 249–256.

Panagiotidis D, Clemens B, Habel U, Schneider F, Schneider I, Wagels L, *et al* (2017). Exogenous testosterone in a non-social provocation paradigm potentiates anger but not behavioral aggression. *Eur Neuropsychopharmacol* doi:10.1016/j.euroneuro.2017.07.006.

Schneider F, Gur RC, Gur RE, Muenz LR (1994). Standardized mood induction with happy and sad facial expressions. *Psychiatry Res* **51**: 19–31.

Wagels L, Radke S, Goerlich KS, Habel U, Votinov M (2017). Exogenous testosterone decreases men’s personal distance in a social threat context. *Horm Behav* **90**: 75–83.
