# Supplementary figures and images for: Exogenous Testosterone Enhances the Reactivity to Social Provocation in Males
Source: Front Behav Neurosci. 2018 Mar 2;12:37. doi: 10.3389/fnbeh.2018.00037 (PMC5840258; doi:10.3389/fnbeh.2018.00037)

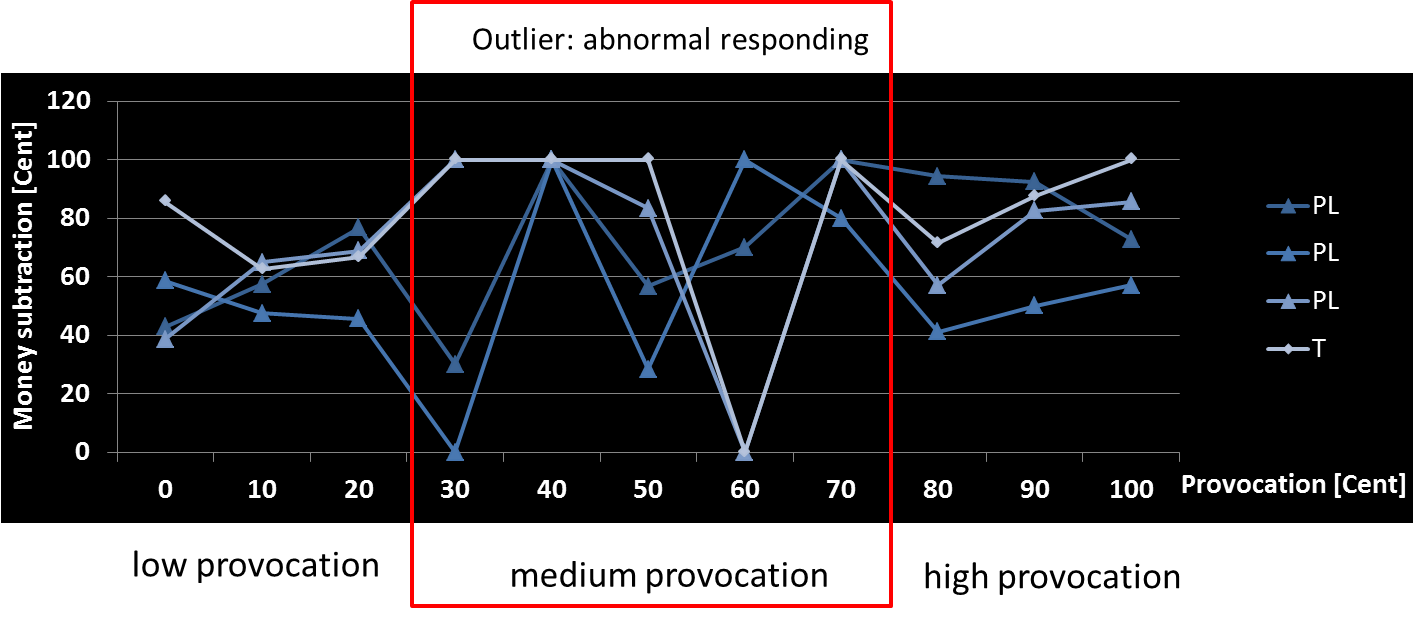

Supplement: FIGURE S1 — Mean values of aggressiveness separated for preceding provocation levels for the four outliers. Abnormal responding is mainly shown after medium provocation (indicated within the red frame). [file Image_1.TIF]
